# Seismic design of setback irregular steel structures based on life cycle cost

**DOI:** 10.1038/s41598-022-21247-8

**Published:** 2022-10-06

**Authors:** Sajad Taheri Jebelli, Behrouz Behnam, Payam Tehrani

**Affiliations:** School of Civil and Environmental Engineering, Amirkabir University of Technology, Tehran, Iran

**Keywords:** Natural hazards, Engineering

## Abstract

The seismic design of conventional structures is mainly addressed considering the direct construction cost; the life cycle costs (LCCs) are often neglected. This paper proposes a performance-based framework for optimal seismic design of irregular steel structures; the LCC is involved as an optimization criterion. Two regular 7- and 10-story structures are first designed based on the design earthquake; their geometries are then changed to make them setback irregular having in overall four cases to investigate. Nonlinear analyses are performed to estimate the target displacement for annual exceedance probabilities, different specified acceleration levels, and, accordingly, the extent of the structural damage. The LCCs of the cases studied are calculated to achieve two objectives: an LCC-based optimal design of steel structures, and evaluating the extent of irregularity on the structures’ LCCs. Results indicate that in the regular and irregular 7-story structures, a 40% and a 50% increase in the seismic loads can respectively reduce the LCCs by 31.3% and 34.9%. In the same vein, in the 10-story regular and irregular structures, increasing the seismic loads by 50% can reduce the LCCs by 33.4% and 31.7%, respectively. The results highlight the point that irregular structures, overall, require a higher initial cost than regular structures when the LCC is taken into account as an optimization criterion.

## Introduction

Regarding the earthquake disaster management cycle, including prevention, preparation, response, and recovery^1^, taking appropriate measures to reduce related damages is inevitable. The prevention activities mitigate or eliminate the possibility of disaster or decrease the impacts of inevitable events. Meanwhile, the whole disaster management cycle includes developing policies and programs to correct the causes of disasters or reduce their impacts on people, property, and infrastructure^1^. Amending and improving the building design regulations and guidelines can significantly contribute to disaster prevention. To this end, the costs induced by natural disasters such as earthquakes can play a major role in the life cycle cost (LCC) of structures; this is specifically the case for irregular tall buildings that are even further prone to sustain damage. Thus, considering these costs may lead to risk reduction policies in the life cycle.

Many studies have shown that a large portion of the LCC of a project relies on the operation phase. For example, Flanagan et al.^2^ showed that the investment costs of a building encompass half the total life costs. Galibourg^3^ argued that the operating costs of a commercial building account for 75% of the LCC (excluding the land acquisition costs). Huang et al.^4^ concluded that the maintenance and operation costs of the studied systems were 73.7–83.9% of the LCC.

The effect of maintenance and operation costs is so significant that any effort to neglect them would be a major loss for customers and the professional competence of the design and construction teams^5^. The investors of durable buildings have also realized that a small increase in the initial costs can significantly reduce the building costs in the future. Thus, it can be concluded that the decisions merely based on the acquisition cost may not be the best choice in the long run. Therefore, the beneficial implications of long-term expenses can be effectively realized using LCC-based methods^6^. It can also be used as a proper evaluation tool for designing sustainable buildings^7^.

**Figure 1:**
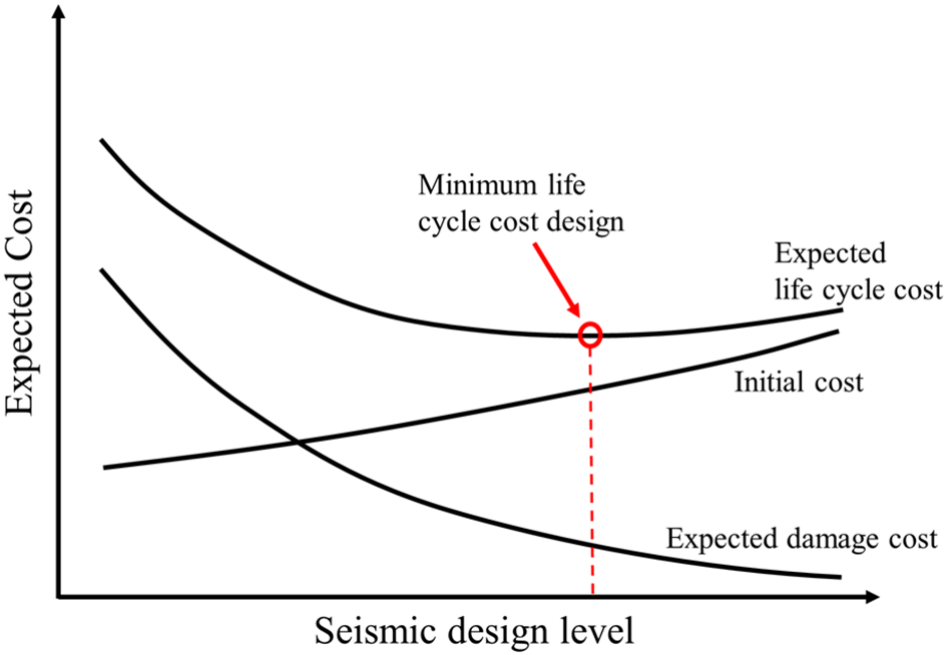
Illustration of the expected life cycle cost^10^.

Due to the major damages caused by the large earthquakes during the 1990s in US and Japan, the engineering community began to question the efficacy of seismic design codes. Despite the low number of human causalities, the financial damages were significant^8^ indicating that although "life safety" is the main design criterion, it should not be the only design criterion. Therefore, it can be argued that design based on current seismic regulations is no longer the best and optimal design, even in terms of financial matters; the LCC-based design methods can be an alternative. In general, LCC decisions require considering the structural costs and natural disasters during the structure's lifetime. Ideally, eliminating the seismic damage risk can eliminate both casualties and financial damages. Although robust and adequate, reliable structures, which resist rare seismic loads without any severe damages, can be theoretically constructed by adopting available technologies^9^, such structures would incur a great initial cost. Hence, the proper selection of the design load is not merely a safety issue. The optimal decision would balance the cost and revenue of a design. Figure 1^10^ shows that an optimal design point for project LCC should be sought out.

Nevertheless, there are some challenges in developing an LCC-based procedure for a design purpose such as the uncertainties, structural limit states, potentially big losses, and the balance between losses and the cost of structural strength^11^. In this context, the LCC analysis has become a fundamental component of the structural design process by considering performance criteria and a robust tool for evaluating the investment and quantitative risk assessment, e.g., to evaluate the losses caused by earthquake events, to control the initial and future costs of buildings^12,13^. In this regard, emphasis has been put on the necessity of optimal structural design in the 1970s; e.g. Rosenblueth and Mendoza^14^, Hasofer^15^, and Liu et al.^16^). Then, scholars, e.g., Rosenblueth and Jara^17^, Rackwitz^18^, Liu et al.^19^, and Goda and Hong^20^, began to develop optimal design methods to select seismic design levels and establish some guidelines. Liu et al.^21^ proposed a multi-objective optimization method based on a genetic algorithm for steel moment frame structures considering the weight, maximum inter-floor relative displacement for performance level, and design complexity criteria. Sarma and Adeli^22^ solved a multi-criteria optimization problem to optimize the LCC of steel structures. Li and Cheng^23^ presented a damage reduction technique as a structural optimization problem. They showed that the proposed method would lead to better seismic designs regarding LCC criteria and maximum relative displacement between two floors. Gencturk^24^ analyzed the LCC of reinforced concrete and composite frames. Taflanidis and Gidaris^25^ proposed a systematic probabilistic framework for accurate estimation and optimization of LCC using dampers to mitigate the seismic risk of building structural systems. They also studied probabilistic approaches for the economical design of viscous dampers based on LCC. Shin and Singh^26^ developed a method to calculate the damages and life cycle costs of various levels of damage considering random seismic events and the uncertainty of the calculated response. Nour Eldin et al.^13^ compared the LCC of reinforcing steel structures using hybrid and steel slit dampers. Behnam^9^ compared the LCC of three steel moment frames with 4, 7, and 10 stories under various seismic loading. He demonstrated that an increase of 60%, 50%, and 40% in the seismic design loading for the 4, 7, and 10-story frames, respectively, would yield a minimum LCC. Hassani et al.^27^ conducted similar studies on concrete moment resisting frames.

Despite the great body of literature regarding LCC-based seismic design of structures, there are no studies concerning the architectural aspects such as regularity and irregularity. Many studies have proved the distinction between the seismic behavior of regular and irregular buildings, but few studies- if any- have addressed the LCC of irregular structures. Last earthquakes have revealed that irregular buildings require special attention because architectural irregularity can significantly increase earthquake damages. Reports on previous earthquakes and literature illustrate that, on average, irregular structures are more susceptible to damage than their regular counterparts^28^. For instance, Shojaei and Behnam^29^ compared three irregularities of setback, soft story, and short column, with regular buildings and found that the highest damage index corresponds to the soft story structure. Therefore, the buildings' geometry can significantly affect the seismic resistance.

This paper aims to account for the LCC of setback irregular buildings, as one of the most common architectural irregularities in urban regions; this is worth mentioning that quantifying the possible damage cost which might sustain by irregular structures over an earthquake is of particular importance when it is compared with regular structures. The work here is to highlight this difference from a performance-based viewpoint.

## Optimal seismic design procedure

The theories and methods used in this study for structural design, performance evaluation, and LCC analysis are described in this section. Generally, the costs were estimated based on the indexes related to the economic situation in Iran.

### Performance-based design

**Figure 2:**
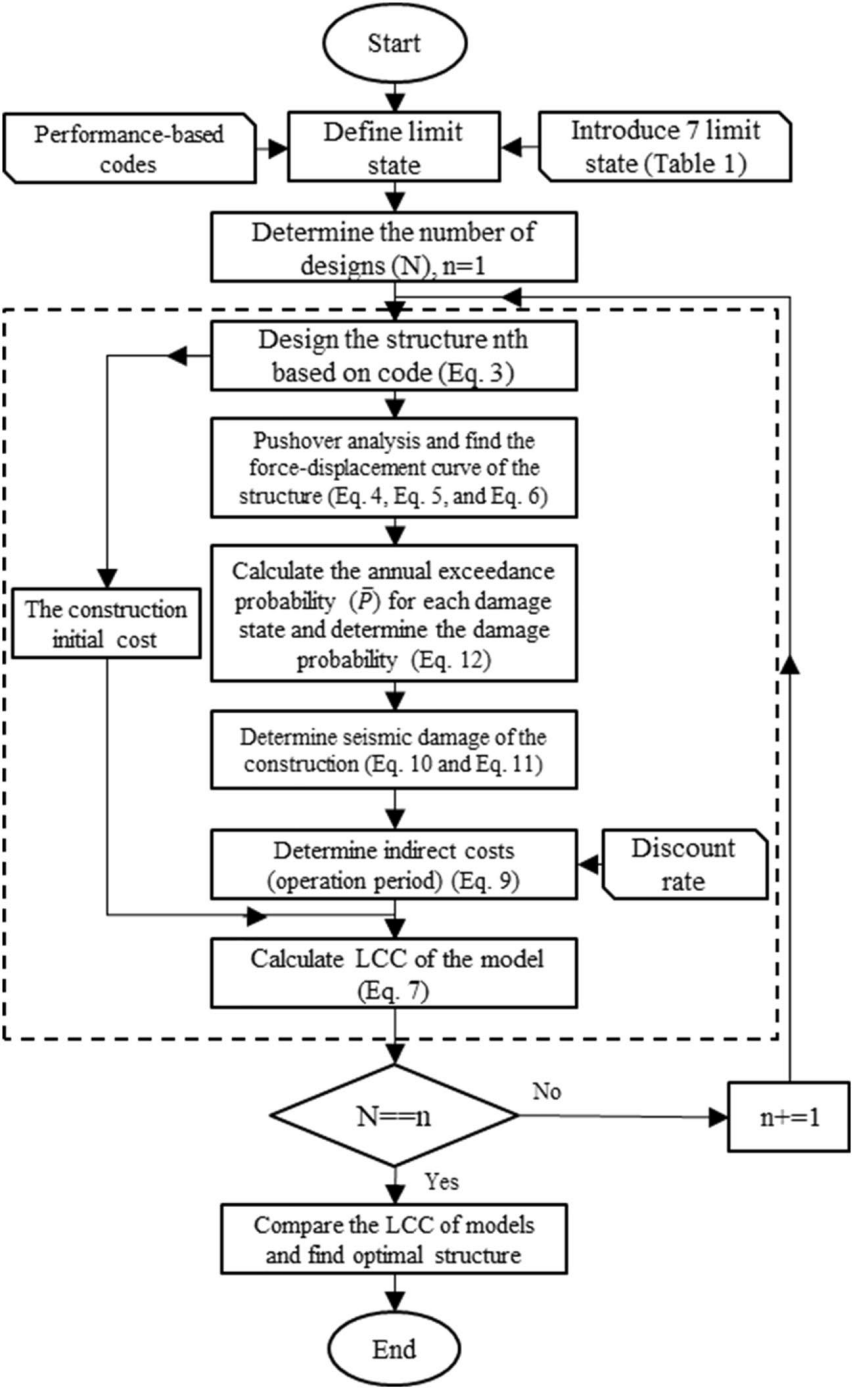
Flowchart of the optimization procedure.

The methodology employed here is summarized in Fig. 2, where the first step is to define the structure limit states where the structure performance levels are defined. The performance level is independent of earthquake intensity, but it correlates with structure usage.

Regarding the performance-based design criteria, the structural performance is controlled by attributing a performance level based on the seismic classification in terms of building usage, earthquake relative consequences, and the importance of earthquake damage mitigation and safety enhancement. This means that the structure is not region-specific, and they are distributed in low-risk and high-risk regions. According to FEMA 450, three earthquakes with 50, 10, and 2% exceedance probabilities for 50 years were selected for three IO, LS, and CP performance level^30^. Annual exceedance probability (*P*) for an earthquake with the exceedance probability, *p*, in *t* years is estimated using Eq. (1).

1 $$ P = \frac{ - 1}{t} {\text{ln}}\left( {1 - p} \right) $$

The annual exceedance probability is proportional to the inverse return period. Thus, it is required to determine the equivalent spectral acceleration (SA) to calculate the base shear induced by an earthquake. The equivalent SA with the exceedance probability of 10% in 50 years is often called "design SA". The SA of earthquakes with the exceedance probabilities of 2% and 5% in 50 years are determined by scaling up the design SA. The scaling is performed using hazard curves, which are plots of the annual frequency of exceedance versus spectral accelerations at different periods (Sa(Ti)). The location of a building determines which hazard curve should be used. Consequently, using hazard curves of different cities for a building leads to different results. However, our proposed framework for building optimization does not rely on the cases studied.

**Figure 3:**
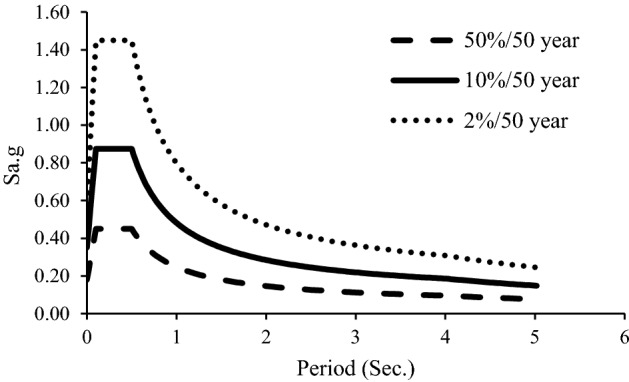
Spectrum accelerations of 2%, 10%, and 50% probability of exceedance over 50 years for Tehran with the soil of Type II (Vs = 375–750 m/s).

The proposed framework was used for residential buildings located in Tehran, Iran. According to FEMA 450, the expected performance level for earthquakes with 50, 10, and 2% exceedance probabilities in 50 years are IO, LS, and CP, respectively. According to Iran's seismological code, the PGA for Tehran's design earthquake is 0.35 g. Based on the hazard curve proposed by Tsang et al. for Tehran using previous events and simulations^31^, the PGA for an earthquake with the annual exceedance probability (AEP) of 2475^–1^ and 72^–1^ are 0.58 and 0.18, respectively. Calculating the ratio of these PGAs to the PGA resulting from the design earthquake, the equivalent SA for the seismic hazard level is compared in Fig. 3. The target displacement for each AEP, i.e., each specified acceleration level, was determined using the generated spectrums.

The damage probability is often described using the inter-story drifts sustained by a structure. This is because more severe drifts lead to higher levels of damage. For most performance-based codes, the inter-story drifts are related to the various levels of performance and damage. For example, ATC-13 suggests seven levels of damage corresponding to different drifts, as listed in Table 1. For drifts lower than 0.2%, it is assumed that no failure would occur, whereas more than 5% drift leads to collapse. The damage caused by drifts in this range is described as minor to major.

**Table 1:** Damage states, maximum inter-story drift limits, and indirect cost^37^.

Performance level	Damage state	Inter-story drift (%)	Indirect cost (% of the initial cost)
1	None	∆ < 0.2	0
2	Slight	0.2 < ∆ < 0.5	0.5
3	Light	0.5 < ∆ < 0.7	5
4	Moderate	0.7 < ∆ < 1.5	20
5	Heavy	1.5 < ∆ < 2.5	45
6	Major	2.5 < ∆ < 5.0	80
7	Destroyed	5.0 < ∆	100

Structures designed based on O and IO performance levels might experience up to 1% transient drift, but no permanent drifts are allowed. Structures designed based on LS and CP performance levels would experience up to 2 and 4% drifts, respectively. It is worth mentioning that there are some probabilistic methods to estimate the seismic performance of a structure called "loss assessment"^32^. The Pacific Earthquake Engineering Research (PEER) Center proposed Eq. (2), where the seismic performance is related to probabilistic earthquake intensity models, demand, and damage^33^.

2 $$ \lambda (DV > dv) = \iiint {G\left( {dv{|}dm} \right)}dG\left( {dm{|}edp} \right)dG\left( {edp{|}im} \right)\left| {d\lambda \left( {im} \right)} \right| $$

where *im* is the spectral acceleration, *edp* is a numerical demand (e.g., inter-story drift), *dm* is the level of damage, and *dv* is a decisive variable (e.g. cost). After choosing the performance level of the structure, the method continues to model the structure.

### Seismic loading and optimization constraints

We should first make sure that the structure will satisfy the design requirements such as the column-to-beam strength ratio, which is known as the "strong column-weak beam" design philosophy. In addition to these limitations, all AISC checks should be met for gravity loads to perform the pushover analysis. According to ASCE-7, gravity load combinations are as follows:

3 $$ Q_{D} = 1.2Q_{D} + 1.6Q_{L} $$

If the constraints are satisfied, the pushover analysis is performed. Here, the gravity load combinations, according to ASCE41-17^35^, are as follows:

4 $$ Q_{D} = 1.1\left( {Q_{D} + Q_{L} } \right) $$

5 $$ Q_{D} = 0.9Q_{D} $$

Between the above combinations, whichever creates a more critical effect on the structure is considered.

### Nonlinear static analysis

It is common knowledge that the criteria of seismic design regulations are based on "resistance-based design" using linear static and dynamic analysis (e.g., response spectrum analysis). These methods assume that all structural elements behave linearly. However, most structural elements behave nonlinearly during a severe earthquake. Therefore, it is required to adopt the nonlinear analysis of structures to achieve accurate solutions, especially for irregular structures, and to represent the seismic responses of the structure better. Although the nonlinear dynamic analysis method is known to be the most accurate approach to evaluating a structure's needs, it is not possible to widely use such a method due to practical issues, modeling limitations, and complex and time-consuming calculations. The pushover analysis provides a good approximation of structural behavior, using a simple modeling procedure with simple calculations in a short time. Thus, the pushover analysis was performed to evaluate the seismic performance of the structures in the current study.

The pushover analysis aims to assess the structural performance in terms of resistance and deformation capacity at the global and element levels. The model is pushed using a fixed, predetermined lateral load pattern. The pushover analysis assumes that the structure's response corresponds to the response of a single-degree-of-freedom system with features similar to the first mode of the structure. However, some efforts were made to consider the effect of higher modes, e.g., Chintanapakdee and Chopra^34^. The pushover analysis applies the seismic loads incrementally while the structure is constantly under gravitational loading. As soon as reaching the target displacement, if the algorithm could not converge, the analysis is terminated. For this study, the pushover analysis requirements such as the prediction of target displacement are based on ASCE41-17^35^, as follows:

6 $$ \delta_{t} = C_{0} C_{1} C_{2} S_{a} \frac{{T_{e}^{2} }}{{4\pi^{2} }}g $$

where C_0_, C_1_, and C_2_ are modification factors. T_e_ is the effective fundamental period of the structure in the direction under consideration. S_a_ is the response spectrum acceleration corresponding to the T_e_ period, normalized by g.

The design and pushover analysis were performed using ETABS 18. Using lumped plasticity, the potential locations of plastic hinges were defined as at 0.05 and 0.95 from beams and columns. As Inel and Ozmen^36^ showed that it is reasonable to use capacity curves for default hinges models in analytics software for buildings compatible with new codes, we used the default settings conforming to ASCE41-17 for the plastic hinges^35^.

### Life cycle cost

An LCC comprises direct cost (C_D_) and indirect cost (C_ID_), meaning the total monetary values over the life of a structure^9^. According to Eq. (7), the direct cost includes construction cost (C_c_), maintenance cost (C_m_), and disposal cost (C_d_)^9^. In this study, the maintenance and disposal costs were neglected.

7 $$ C_{D} = C_{c} + C_{m} + C_{d} $$

The initial cost of steel structures is usually proportional to the total weight of the components. Parameters affecting the initial cost, such as non-structural components cost and fire and corrosion protective covers were also neglected.

The only considered indirect LCC here is the cost of potential earthquake damage during the structure's lifetime. As shown in Eq. (8), C_ID_ includes repairs (C_re_), loss of possessions (C_p_), relocation (C_r_), economic loss (C_e_), casualties (C_cas_), and fatalities (C_f_) costs. Here, we assume that C_re_ is only for one building, and the infrastructure damage does not directly affect C_re_. Some codes such as ATC-13^37^ have estimated the average time required to repair the damaged structures. This average time depends on the number of stories and the level of damage.

8 $$ C_{ID} = C_{re} + C_{p} + C_{r} + C_{e} + C_{cas} + C_{f} $$

On the one hand, when the damage is significant, the residents may have to leave the building and rent a new place during the repair time, which delays the building's performance and incurs additional expenses for the residents; this is especially the case in high-rise buildings. As well, human injuries and casualties are a big loss for society because today's economists assert that the workforce is a critical national resource that makes the return on investment possible. Factors such as expertise, ability, and knowledge that require training programs can be used as a resource in any economic activity. Since the expense of such training programs is provided by the society, the costs are expected to be reimbursed after a while, e.g., within 30 years of working life. Accordingly, if a person leaves the cycle sooner than the desired period, e.g., due to an accident or illness, this causes a socioeconomic disadvantage with both implicit and explicit consequences^9^. Determining the level of economic losses imposed by these factors relies on multiple socioeconomic criteria. For example, several approaches calculate the cost of loss of life, including economic approaches and those that consider the loss of life irrecoverable^38^. Yet, the cost estimation of exceeding the CP limit state differs from the approach adopted in the current study.

Several probabilistic methods have been proposed to calculate LCC, one of which is the function proposed by Wen and Kang^11^ as given in Eq. (9). The indirect earthquake cost was calculated as a percentage of the initial cost reported in Table ATC-13^37^ (the second column of Table 1) and the proposed method by Fragiadakis et al.^8^, detailed in section “Conclusions”.

9 $$ E\left[ {C\left( {t.X} \right)} \right] = C_{0} + \left( {C_{1} P_{1} + C_{2} P_{2} + \cdots + C_{k} P_{k} } \right)\frac{v}{\lambda }\left( {1 - e^{ - \lambda t} } \right) $$

where P_k_ is the kth probability of the limit state considering the earthquake event and C_k_ is the corresponding cost (the fourth column of Table 1).

10 $$ P_{k} = P_{k} \left( {\Delta > \Delta_{k} } \right) - P_{k + 1} \left( {\Delta > \Delta_{k + 1} } \right) $$

11 $$ P_{k} \left( {\Delta > \Delta_{k} } \right) = \left( {\frac{ - 1}{t}} \right){\text{ln}}\left[ {1 - \overline{{P_{k} }} \left( {\Delta > \Delta_{k} } \right)} \right] $$

where $\overline{{P_{k} }} \left( {\Delta > \Delta_{k} } \right)$ is the annual exceedance probability of maximum inter-story drift, v is the annual occurrence rate of major earthquakes, modeled using the Poisson process, and t is the service life of a new structure or the remaining service life of a retrofitted structure. The service life of structures was assumed to be 50 years. C_k_ would be incurred on the structures in the future while the construction costs are current. This means that all indirect costs should be classified such that their results prove their actual value at the construction time. Therefore, the exponent in the equation was used to represent C_ID_ in the current value. Thus, the annual discount rate (λ) was considered a constant number of 5% which is within the Asian discount rate (5–8%)^39^. It was assumed that the structure could fully recover its pre-earthquake state^8^.

Each damage state corresponds to maximum inter-story drifts as listed in Table 1 (column 3); when the drift exceeds the values, it reaches its corresponding limit state. The annual exceedance probability $\overline{{P_{k} }} (\Delta > \Delta_{k} )$ for the damage state is then calculated using Eq. (12).

12 $$ \overline{{P_{k} }} \left( {\Delta > \Delta_{k} } \right) = {\upalpha }e^{{ - \beta \Delta_{k} }} $$

where α and β are determined using the best graph fitted with the known pair values of $\overline{{P_{k} }} - \Delta_{k}$. These pairs were determined by the response of earthquakes with the exceedance probabilities of 2, 10, and 50% in 50 years. The AEP with the exceedance probability p in t years is calculated by Eq. (1). For example, the 10/50 earquake has an AEP of $\overline{P}_{10\% } = \left( {\frac{ - 1}{{50}}} \right).\ln \left( {1 - 0.1} \right) = 2.107 \times 10^{ - 3}$.

## Numerical study

**Figure 4:**
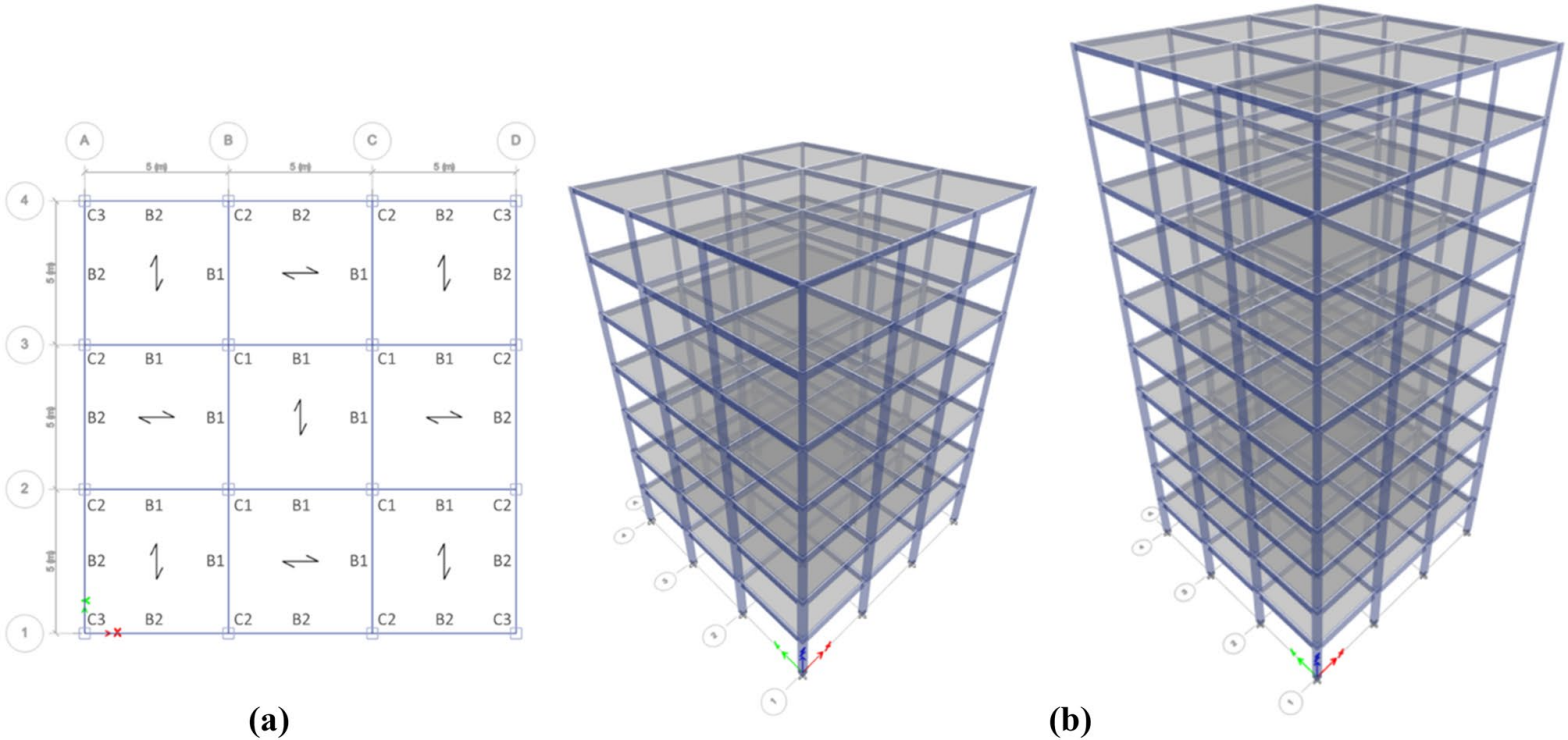
The (**a**) structural plan and (**b**) views of the regular 7 and 10-story structures.

**Figure 5:**
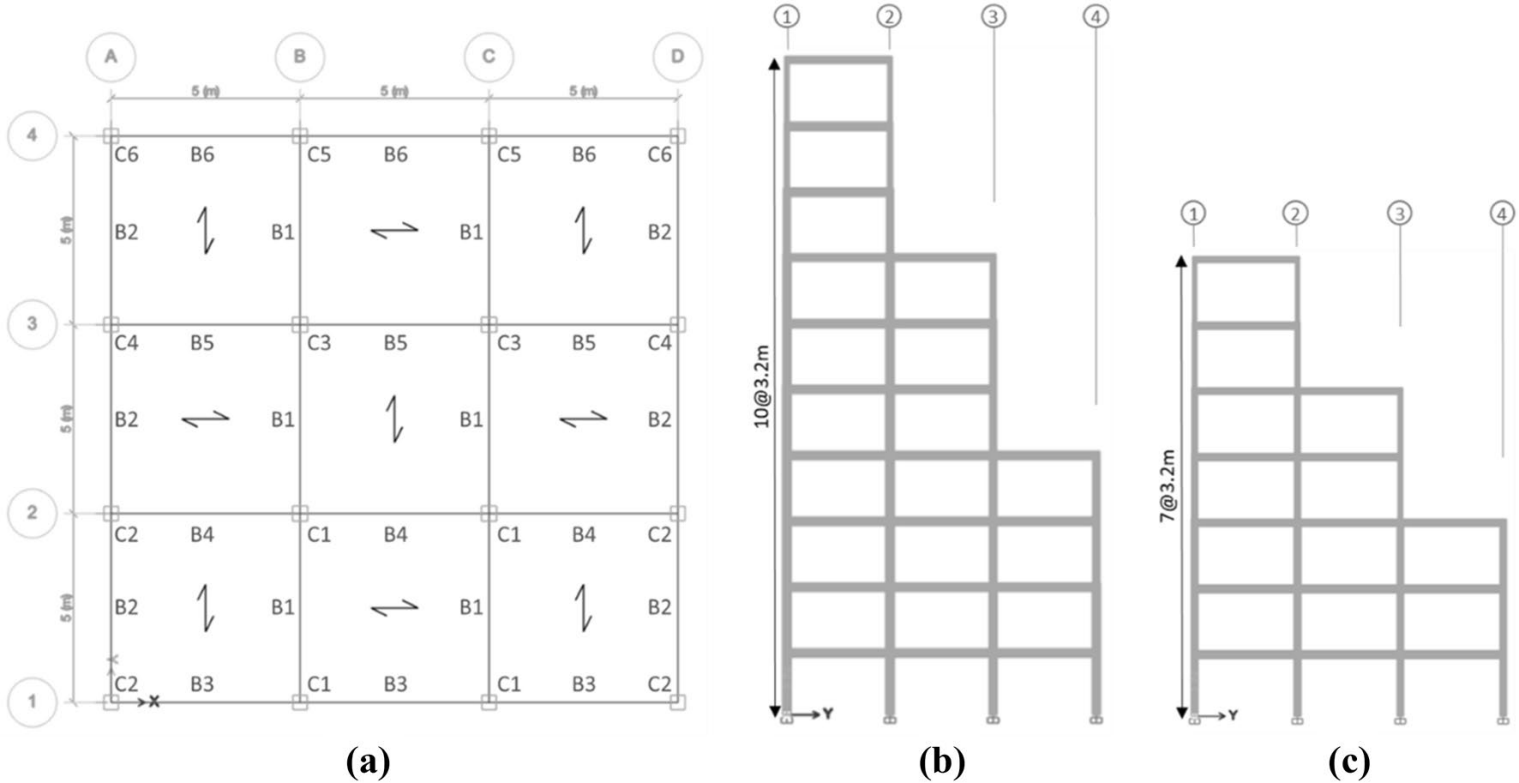
(**a**) The structural plan and sections of the setback irregular (**b**) 10 and (**c**) 7-story structures.

The methodology described in the previous section is now employed to determine and compare the optimal LCC of four regular and irregular steel moment structures with 7 and 10 stories. As indicated in Fig. 4, three equal 5-m spans are modeled in both directions. The story height is 3.2 m, making the whole structure 22.4 and 32.0 m long for the 7- and 10-story structures, respectively. Two setback irregular structures with the same number of stories, spans, and heights are considered the regular ones, as shown in Fig. 5. The horizontal dimensions of one story in a setback irregular structure are 130% larger than that of the adjacent stories^40^. Therefore, when the horizontal dimension difference for two adjacent stories in the modeled structures along the y-axis is 5 m, the setback irregularity condition is satisfied. The structures are supposed to be located in Tehran, Iran. According to the Iranian National Building Code- Part 6 (INBC6), equivalent to UBC IV (z = 0.4) region and ASCE-07 E seismic region, the design PGA is 0.35 g. According to INBC6, the soil is of Type II, which falls into the ASCE-07 class C. The 7- and 10-story buildings are designed to conform to intermediate and special moment frame regulations. The latter is designated consistent with INBC10, which is very similar to AISC360-05 and AISC341-05. The buildings are considered residential, and according to ASCE41-17, they are assessed to meet the LS performance level. A dead load of 650 kg/m^2^ is applied to all story floors, and a live load of 250 kg/m^2^ is applied to all story floors (except for the roof with a live load of 150 kg/m^2^). The perimeter walls are under a 250 kg/m^2^ load exerted linearly on perimeter beams. The roof of each floor is made of composite with steel decks of 7.5 cm depth, steel with the ultimate strength of 370 MPa, and a 7.85 cm-thick concrete with an ultimate strength of 28 MPa. The composites of the roofs are laid in a checkered position to ensure the uniform distribution of load on structural components. A combination of 100% dead load and 20% live load is used to find the required mass, and consequently, to calculate the seismic load.

The structures are designed to have a tensile strength equal to 240 MPa, the ultimate strength of 370 MPa for steel sections, and compressive strength of 28 MPa for the concrete laid on the steel deck floor. The beams and columns are designed using IPE and hollow-square sections, respectively. The seismic compactness of all sections is controlled according to INBC10. The cross-sectional dimensions of all beams and columns are designed such that the strong column-weak beam principle and component strength can satisfy all controls; although according to INBC10, it is not required for intermediate moment frames. Floors and frame joints were assumed to be rigid and fixed. The sections used in designs are demonstrated in Figs. 4 and 5 as visible types for regular and irregular structures, respectively. More details of the selected sections are presented in Table 2.

**Table 2:** Specifications of beams and columns (The first value is the section’s width and the second value is the section’s thickness (in mm)).

Case	Section	Story									
		1	2	3	4	5	6	7	8	9	10
7 Story—regular	C1	400X20	400X20	400X20	340X20	300X20	260X20	220X15			
	C2	380X20	380X20	380X20	320X20	320X20	260X20	220X15			
	C3	380X20	380X20	320X20	260X20	260X20	200X12	180X10			
	B1	IPE450	IPE450	IPE450	IPE400	IPE360	IPE330	IPE270			
	B2	IPE400	IPE400	IPE400	IPE360	IPE330	IPE330	IPE240			
7 Story—setback irregular	C1	360X20	360X20	360X20	320X20	300X20	260X20	220X20			
	C2	340X20	340X20	340X20	300X20	280X20	240X20	200X15			
	C3	320X20	320X20	320X20	300X20	300X20					
	C4	320X20	320X20	320X20	240X20	240X20					
	C5	320X20	320X20	320X20							
	C6	320X20	320X20	320X20							
	B1	IPE400	IPE400	IPE360	IPE360	IPE360	IPE360	IPE330			
	B2	IPE400	IPE400	IPE360	IPE360	IPE330	IPE360	IPE330			
	B3	IPE400	IPE400	IPE400	IPE400	IPE360	IPE330	IPE270			
	B4	IPE360	IPE360	IPE360	IPE360	IPE330	IPE330	IPE270			
	B5	IPE330	IPE330	IPE330	IPE330	IPE270					
	B6	IPE330	IPE330	IPE330							
10 Story—regular	C1	400X30			380X30	380X25	340X20	320X20	300X20	260X20	200X20
	C2	400X25	400X25	400X25	380X25	380X20	320X20	320X20	280X20	240X20	180X12
	C3	400X25	400X25	380X20	380X20	320X20	280X20	240X20	200X20	200X15	180X10
	B1	IPE500	IPE500	IPE500	IPE450	IPE450	IPE400	IPE360	IPE360	IPE330	IPE270
	B2	IPE500	IPE500	IPE450	IPE450	IPE400	IPE400	IPE360	IPE330	IPE300	IPE270
10 Story—setback irregular	C1	420X25	420X25	420X25	400X25	400X25	380X20	380X20	340X20	300X20	220X20
	C2	400X25	400X25	400X25	400X20	400X20	380X20	380X20	320X20	240X20	200X20
	C3	380X20	380X20	360X20	360X20	340X20	340X20	300X20			
	C4	380X20	380X20	340X20	340X20	320X20	320X20	260X20			
	C5	380X20	380X20	360X20	360X20						
	C6	380X20	380X20	340X20	340X20						
	B1	IPE400	IPE400	IPE400	IPE400	IPE400	IPE450	IPE360	IPE450	IPE400	IPE330
	B2	IPE400	IPE400	IPE400	IPE400	IPE400	IPE400	IPE360	IPE400	IPE400	IPE330
	B3	IPE450	IPE450	IPE450	IPE450	IPE450	IPE400	IPE400	IPE360	IPE330	IPE270
	B4	IPE450	IPE450	IPE450	IPE400	IPE400	IPE400	IPE330	IPE330	IPE300	IPE270
	B5	IPE360	IPE360	IPE360	IPE360	IPE360	IPE360	IPE300			
	B6	IPE360	IPE360	IPE360	IPE360						

It is well documented that the term optimization is referred to as a process via which perfect results are obtained in a system from different views as such cost or time. While obtaining such results are not practically possible due to real limitations, we should hence modify an optimization process to a level it can be accepted. We here consider an LCC as optimized in which by increasing the shear forces applied to the cases studied it does not change significantly.

All components are now re-designed by changing the seismic loads to determine the optimum LCC for each structure. The seismic loads are first increased by 10% in a step-wise manner; overall, six models are re-designed in a way that the last model is designed for 1.6 times the design earthquake. Two models are then re-designed by a 10% decrease in each step so that the second model is re-designed based on an 80% design earthquake. In general, nine models are created based on various seismic loads; one is based on the design earthquake, and the rest for different seismic loads as defined above. The models are named based on xF_Yi where F stands for the seismic load, x is the increase or decrease coefficient applied to the design earthquake, and Y is the structural type (i.e., R = regular and IR = irregular), and i is the number of stories. For instance, 0.8F_R7 is a regular 7-story structure designed for the 20% decreased design earthquake, and 1.3F_IR10 is an irregular 10-story structure designed for the 30% increased design earthquake.

As discussed earlier, LCCs include direct and indirect costs. The direct cost consists of the initial construction cost. Here, the construction cost data of 270 buildings in Tehran, including the cost of structural and non-structural components, were gathered. It was found that the structural cost accounted for around 9% of the total construction costs on average. Thereby, the cost of non-structural components can be predicted by estimating the total construction cost based on the structural cost. It is obvious that any change in the seismic loading only affects the structural cost. The gathered information also indicated that depending on the building location, the construction cost of a 7- and 10-story structure, using high-quality materials, were 356 and 382 USD/m^2^, respectively. Hence, the total cost of construction (excluding the steel structure cost) was 324 and 348 USD/m^2^. The structural cost can be calculated using a 0.70 USD/kg rate for steel sections.

**Figure 6:**
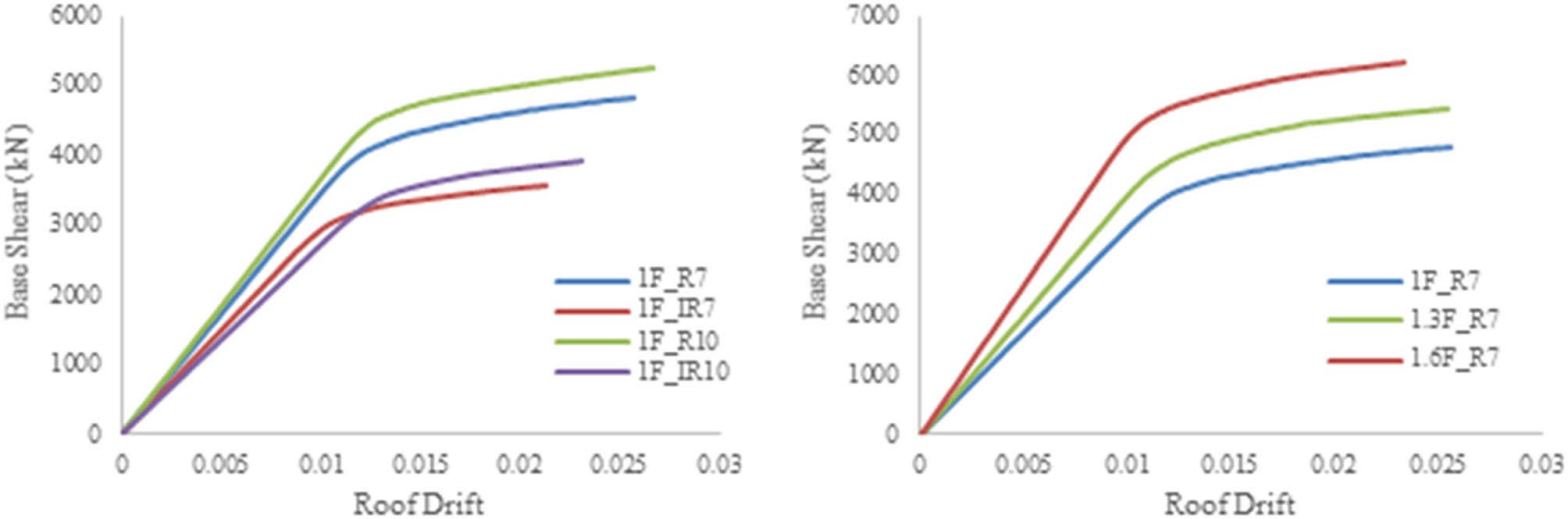
Samples of pushover curves.

The required information to estimate the LCC of a model was described in section “Numerical study”. All design cases are initially designed through the design earthquake and then redesigned for various seismic loadings (i.e., six models for higher seismic loadings and two models for lower seismic loadings). The next step is to carry out the pushover analysis to find the load–displacement curve for both buildings. The lateral loads are applied in a displacement-controlled manner in 100 incremental steps until the loads reached the target displacement for each structure, according to ASCE41-17. Afterward, the pushover curve is obtained for each structure. The target displacement for the three AEP (i.e., PGA of 0.58, 0.35, and 0.18 g) is determined for the earthquakes with the exceedance probabilities of 2, 10, and 50% in 50 years. For instance, the pushover curves for three types of 7-story regular structures and the structures designed based on the design earthquake of all four types are presented in Fig. 6.

**Figure 7:**
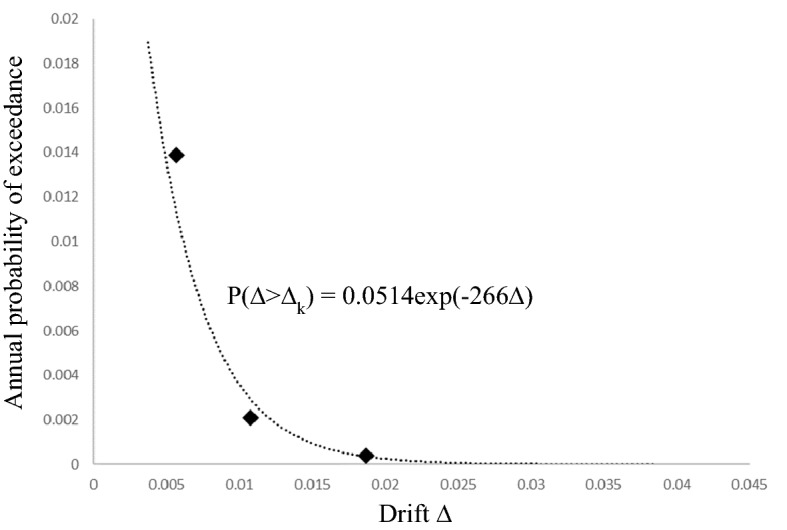
Calculation of annual exceedance probability ($\overline{P }$) for each damage state (Case 1.4F_IR7).

Figure 7 and Table 3 describe the objective function of LCC for the 1.4F_IR7 model. The objective function for the model is described as follows: the pushover analysis result provides three pairs of maximum inter-story drift and AEP (Δ, P): (0.005654, 0.0139), (0.011845, 0.0021), and (0.020939, 0.000404). These pairs correspond to three risk levels with the specified AEP. An exponential function is derived using regression analysis as shown in Fig. 7. The derived function is used to calculate the annual exceedance probability, P, of the seven damage states listed in Table 1. The P values are substituted into the Eqs. (6) and (7) to calculate the LCC value as a function of the initial cost. As an illustration, all the calculations for 1.4F_IR7 are listed in Table 3.

**Table 3:** Calculation of LCC for case 1.4F_IR7.

Damage state	Drift ∆_k_	$\overline{{P }_{k}}\left(\Delta >{\Delta }_{k}\right)$	${P}_{k}\left(\Delta >{\Delta }_{k}\right)$	${P}_{k+1}(\Delta >{\Delta }_{k+1})$	${P}_{k}$	Cost/C_IC_	${C}_{LC}^{k}$/C_IC_
1	0	0.051400	− 0.052768	− 0.030659	0.022109	0	0.000000
2	0.002	0.030194	− 0.030659	− 0.013687	0.016972	0.5	0.008486
3	0.005	0.013594	− 0.013687	− 0.008018	0.005670	5	0.028349
4	0.007	0.007986	− 0.008018	− 0.000951	0.007066	20	0.141326
5	0.015	0.000951	− 0.000951	− 0.000067	0.000885	45	0.039817
6	0.025	0.000067	− 0.000067	0.000000	0.000066	80	0.005314
7	0.05	0.000000	0.000000	0.000000	0.000000	100	0.000009
Sum							0.223301
$\frac{v}{\lambda }(1-{e}^{-\lambda t})$							18.3583
${C}_{LC}$/C_IC_							4.0994

**Figure 8:**
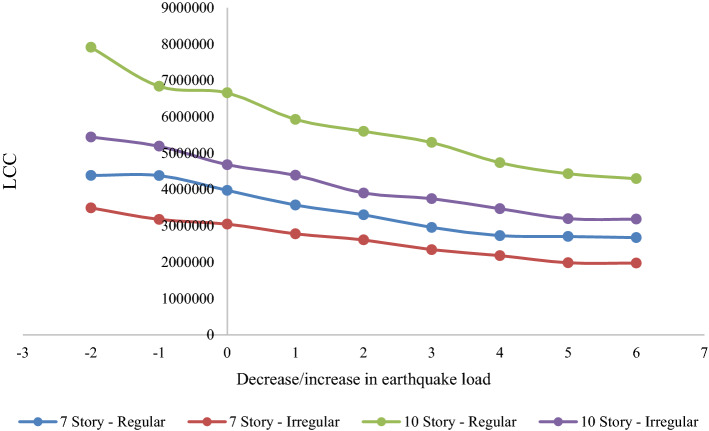
LCCs of the models based on different seismic design loads.

Tables 4, 5, 6 and 7 present the structural results including the total weight of steel sections for all cases as well as their initial, indirect, and LCCs. The 1F_Yi represents the results of the design earthquake. Moreover, the LCC of all buildings is plotted in Fig. 8.

**Table 4:** Results of 7-story regular structures under different seismic loads.

Model	Weight of structure (kg)	Initial cost ($)	Indirect cost ($)	Life Cycle cost ($)	Structure period	Roof drift under earthquake 2%/50
0.8F_R7	95,005	576,804	3,809,111	4,385,915	1.587	0.028092
0.9F_R7	101,611	581,427	3,800,349	4,381,776	1.474	0.026007
1F_R7	108,255	586,079	3,388,978	3,975,056	1.330	0.023642
1.1F_R7	109,744	587,121	2,982,789	3,569,910	1.310	0.022384
1.2F_R7	114,075	590,152	2,712,138	3,302,291	1.281	0.021787
1.3F_R7	120,508	594,656	2,359,533	2,954,188	1.248	0.020591
1.4F_R7	120,848	594,893	2,134,894	2,729,787	1.193	0.019363
1.5F_R7	121,479	595,335	2,111,197	2,706,532	1.168	0.01834
1.6F_R7	131,300	602,210	2,072,177	2,674,387	1.138	0.017928

**Table 5:** Results of 7-story irregular structures under different seismic loads.

Model	Weight of structure (kg)	Initial cost ($)	Indirect cost ($)	Life cycle cost ($)	Structure period	Roof drift under earthquake 2%/50
0.8F_IR7	76,696	418,187	3,075,145	3,493,332	1.372	0.027655
0.9F_IR7	78,194	419,236	2,757,014	3,176,250	1.333	0.026099
1F_IR7	81,059	421,241	2,625,856	3,047,097	1.291	0.025508
1.1F_IR7	82,635	422,344	2,357,873	2,780,217	1.243	0.023374
1.2F_IR7	85,969	424,678	2,187,746	2,612,424	1.155	0.022312
1.3F_IR7	88,579	426,505	1,916,764	2,343,270	1.115	0.020939
1.4F_IR7	89,891	427,423	1,752,187	2,179,611	1.084	0.018697
1.5F_IR7	93,908	430,236	1,553,114	1,983,350	1.057	0.018536
1.6F_IR7	96,321	431,925	1,542,952	1,974,877	1.036	0.019313

**Table 6:** Results of 10-story regular structures under different seismic loads.

Model	Weight of structure (kg)	Initial cost ($)	Indirect cost ($)	Life cycle cost ($)	Structure period	Roof drift under earthquake 2%/50
0.8F_R10	153,048	890,133	7,020,150	7,910,283	2.012	0.029323
0.9F_R10	159,455	894,618	5,943,902	6,838,520	1.904	0.027197
1F_R10	180,841	909,589	5,748,633	6,658,222	1.701	0.027013
1.1F_R10	181,483	910,038	5,015,410	5,925,448	1.684	0.025482
1.2F_R10	186,711	913,697	4,684,776	5,598,474	1.638	0.024269
1.3F_R10	191,601	917,121	4,376,178	5,293,298	1.607	0.021935
1.4F_R10	196,951	920,866	3,814,368	4,735,234	1.570	0.020583
1.5F_R10	203,044	925,131	3,508,020	4,433,151	1.549	0.019829
1.6F_R10	209,099	929,369	3,367,214	4,296,583	1.513	0.019562

**Table 7:** Results of 10-story irregular structures under different seismic loads.

Model	Weight of structure (kg)	Initial cost ($)	Indirect cost ($)	Life cycle cost ($)	Structure Period	Roof drift under earthquake 2%/50
0.8F_IR10	127,763	637,534	4,805,139	5,442,673	1.839	0.030574
0.9F_IR10	137,123	644,086	4,545,848	5,189,934	1.763	0.028103
1F_IR10	138,636	645,145	4,034,095	4,679,240	1.703	0.024745
1.1F_IR10	142,570	647,899	3,743,527	4,391,426	1.596	0.023954
1.2F_IR10	144,361	649,153	3,252,387	3,901,540	1.565	0.022391
1.3F_IR10	147,966	651,676	3,094,912	3,746,588	1.511	0.021752
1.4F_IR10	154,795	656,456	2,815,585	3,472,042	1.447	0.019609
1.5F_IR10	158,234	658,863	2,536,481	3,195,344	1.379	0.019439
1.6F_IR10	164,165	663,015	2,520,174	3,183,189	1.345	0.018001

**Figure 9:**
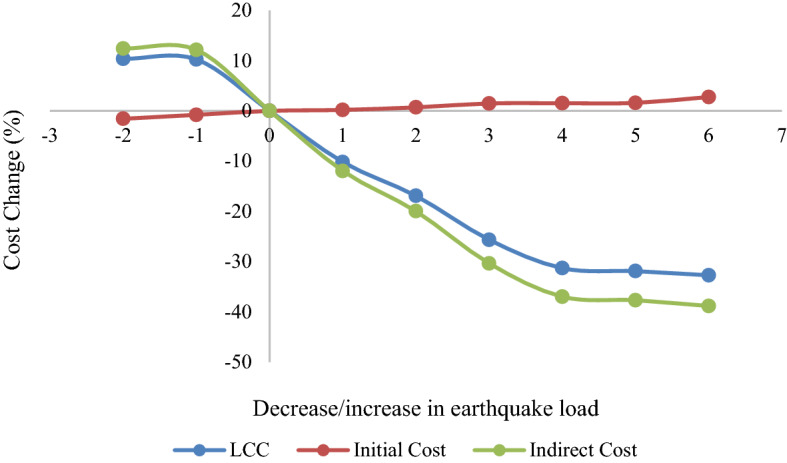
Initial, indirect, and LCCs of the regular 7-story structures under different seismic loads.

As shown in Tables 4, 5, 6 and 7, higher seismic design loads led to higher structure weight and, thus, lower initial costs and lower indirect costs caused by earthquake event probability. As shown in Fig. 9, it is worth noting that for the regular 7-story structure the LCC significantly reduces (higher than 30%) despite the small increase (less than 5%) in the initial cost. It indicates that the LCC is mostly affected by indirect costs rather than direct costs. However, Fig. 8 illustrates that the increment of seismic design loads after a step does not significantly affect the LCC, and increasing the initial cost does not result in lower LCCs. Hence, 1.5F_IR10, 1.5F_R10, 1.5F_IR7, and 1.4F_R7 models can be considered as the LCC-based optimal structures for regular 7-story, irregular 7-story, regular 10-story, and irregular 10-story, respectively.

**Figure 10:**
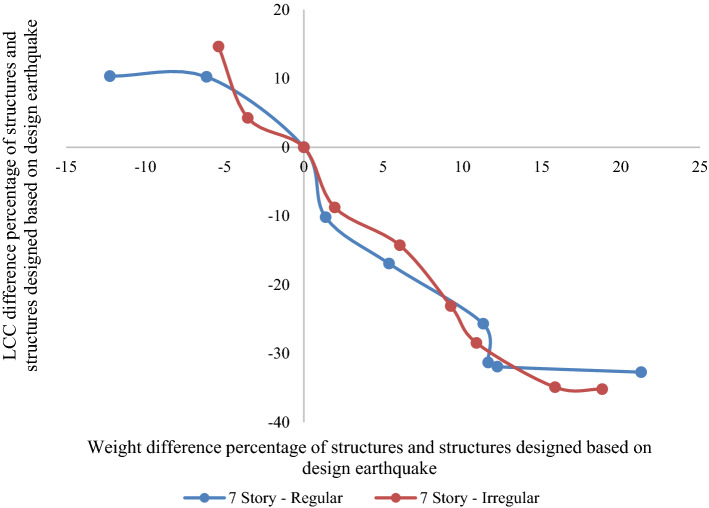
Weight and LCC difference of 7-story models based on the design earthquake (in percentage).

**Figure 11:**
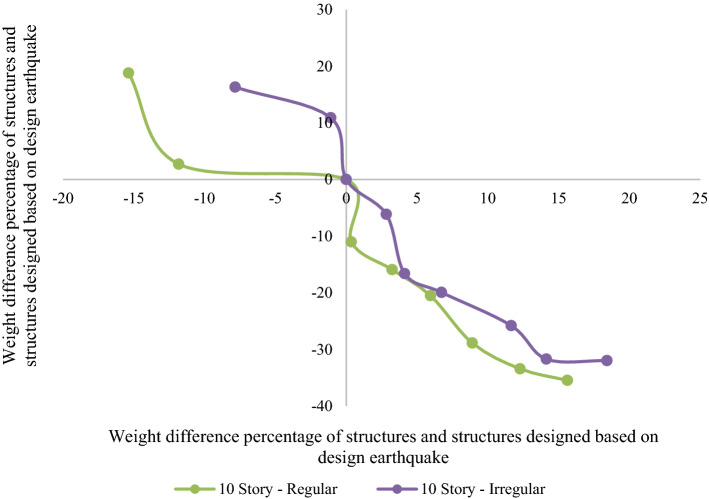
Weight and LCC difference of 10-story models based on the design earthquake (in percentage).

Figures 10 and 11 compare the weight and LCC of each structure with the structure designed using the regulation's design earthquake to assess the effect of irregularity on LCCs. According to Fig. 10, we might not be able to find a specific pattern for the 7-story building. Even though the optimal regular and setback 7-story structures were designed based on a 40% and 50% increase in seismic design load, in the equal situation by an increase of almost 11% in their weights (1.4F_R7 and 1.4F_IR7 models), the LCC of the regular and setback structures reduce by 31% and 28%, respectively. It shows the negative effect of irregularity on seismic performance and the LCC of the structure. As shown in Fig. 11, the effect of irregularity is more obvious for higher structures. Comparing the regular and setback irregular 10-story structures reveals that for equal increment (of the seismic design load), the LCC reduction percentages were lower than the irregular structures, while the weight of all setback irregular structure models was increased more than the regular ones. For example, a 9% weight increase for the 1.4F_R10 model resulted in a 29% reduction in the LCC, whereas a 12% weight increase for the 1.4_IR10 model led to a 26% lower LCC. It indicates that the negative effect of irregularity on structural performance is amplified for structures with more stories.

## Conclusions

For urban seismic areas, earthquakes are always a major concern. A solution addressing such a concern is to design the structures with seismic considerations to provide safety against seismic events. Nevertheless, direct construction costs are always considered when using seismic regulations and the cost of the structure over its service life is often neglected. In this study, a performance-based framework for optimizing steel structures using nonlinear static analysis was adopted to estimate the level of damage caused by various earthquake intensities. This study calculates and compares the life cycle cost (LCC) of irregular and regular structures, considering the different performance of regular and irregular buildings, the higher vulnerability of irregular buildings than regular buildings, and the lack of literature focusing on the LCC of irregular structures.

Four types of structure (2 regular and 2 setback irregular structures with 7 and 10 stories) were assessed to determine the effect of irregularity on the LCC of the structure while using the proposed framework. The evaluations led to the following conclusions:

- For the regular 7-story structure, the LCC of the model designed using the 40% increase in seismic load was reduced by 31.3%.
- For the irregular 7-story structure, the LCC of the model designed using the 50% increase in seismic load was reduced by 34.9%.
- For the regular 10-story structure, the LCC of the model designed using the 50% increase in seismic load was reduced by 33.4%.
- For the irregular 10-story structure, the LCC of the model designed using the 50% increase in seismic load was reduced by 31.7%.

According to this study, when the design aims to minimize the initial weight, the resulting design is more prone to future earthquake damage. Therefore, the total cost of the design during the structure service life would increase. The cost would be specifically higher for irregular and high-rise buildings. It was found that a design using earthquake design regulations would not lead to the optimum LCC. Higher seismic loads can offer far less LCC than earthquake design regulations. This can raise serious questions about the seismic design subject. This difference is even further evident for irregular high-rise structures. Yet, it cannot be concluded that design based on current seismic codes is flawed and that it cannot be criticized regarding their safety for human life during earthquakes. Nevertheless, this study can at least encourage various engineering communities (e.g., manufacturers, designers, and insurance companies) to think differently about the seismic codes.

It was shown how performance-based analysis could be used to optimize a structure, reduce the LCCs, and enhance the strength and resistance of the structure against more severe earthquakes. Based on the proposed method in this study, multiple optimal models with various levels of initial cost and LCC provided diverse options for stakeholders to make the best decision for a project. It should be noted that the proposed framework here is independent of the region; it is applicable to any region provided that the region-specific hazard curves are used. However, the results of the study here are case-specific; thus, they cannot be generalized to other structures worldwide. As well, the results of the study here are based on pushover analysis; the advantages and limitations of the pushover analysis are well documented in many studies. More accurate analyses such as nonlinear dynamic analysis can be pursued in future studies.

## Data Availability

The data that support the findings of this study are available from http://library.aut.ac.ir/. Data are available from the authors upon reasonable request and with permission of http://library.aut.ac.ir; please contact lib_office@aut.ac.ir.
